# Supplementary material for: A new glassfrog (Centrolenidae) from the Chocó-Andean Río Manduriacu Reserve, Ecuador, endangered by mining
Source: PeerJ. 2019 Feb 26;7:e6400. doi: 10.7717/peerj.6400 (PMC6404656; doi:10.7717/peerj.6400)
Supplement: Supplemental Information 1 [file peerj-07-6400-s001.docx]

| **Species** | **Museum number** | **Genbank code (12S)** | **Genbank code (16S)** | **Locality** |
| --- | --- | --- | --- | --- |
| *Nymphargus balionotus* | JMG-0607 | MH746562 | MH746536 | Ecuador: Provincia Imbabura, Reserva Río Manduriacu, 1285 m. |
| *Nymphargus balionotus* | JMG-0796 | MH746563 | MH746537 | Ecuador: Provincia Imbabura, Reserva Río Manduriacu, 1254 m. |
| *Nymphargus balionotus* | JMG-0798 | MH746564 | MH746538 | Ecuador: Provincia Imbabura, Reserva Río Manduriacu, 1240 m. |
| *Nymphargus manduriacu* sp. nov*.* | JMG-0615 | MH746565 | MH746539 | Ecuador: Provincia Imbabura, Reserva Río Manduriacu, 1230 m. |
| *Nymphargus manduriacu* sp. nov*.* | JMG-0616 | MH746566 | MH746540 | Ecuador: Provincia Imbabura, Reserva Río Manduriacu, 1230 m. |
| *Nymphargus manduriacu* sp. nov*.* | JMG-0622 | NA | MH746541 | Ecuador: Provincia Imbabura, Reserva Río Manduriacu, 1242 m. |
| *Nymphargus griffithsi* | MZUTI 100 | MH830303 | MH830299 | Ecuador: Provincia Pichincha, Reserva Las Gralarias, 2175 m |
| *Nymphargus griffithsi* | QCAZ 24824 | MH830306 | MH830302 | Ecuador: Provincia Pichincha, La Victoria, 2104 m |
| *Nymphargus griffithsi* | MZUTI 099 | MH830305 | MH830301 | Ecuador: Provincia Pichincha, Reserva Las Gralarias, 2175 m |
| *Nymphargus griffithsi* | MZUTI 098 | MH830304 | MH830300 | Ecuador: Provincia Pichincha, Reserva Las Gralarias, 2175 m |
| *Nymphargus grandisonae* | QCAZ 16288 | MH746574 | NA | Ecuador: Provincia Pichincha, Quebrada Zapadores, 1900 m. |
| *Nymphargus lasgralarias* | QCAZ 42164 | MH746567 | MH746542 | Ecuador: Provincia Imbabura, San Antonio de Cuellaje, 2720 m |
| *Nymphargus lasgralarias* | MZUTI 095 | MH746568 | MH746543 | Ecuador: Provincia Pichincha, Reserva Las Gralarias, 2200 m |
| *Nymphargus lasgralarias* | MZUTI 097 | MH746570 | MH746545 | Ecuador: Provincia Pichincha, Reserva Las Gralarias, 2175 m |
| *Nymphargus lasgralarias* | QCAZ 46012 | MH746571 | MH746546 | Ecuador: Provincia Pichincha, Nanegal Grande, 2300 m. |
| *Nymphargus lasgralarias* | MZUTI 096 | MH746569 | MH746544 | Ecuador: Provincia Pichincha, Reserva Las Gralarias, 2150 m |
| *Nymphargus lasgralarias* | QCAZ 11689 | MH746572 | MH746547 | Ecuador: Provincia Cotopaxi, Bosque Integral Otonga, 1950 m. |
| *Nymphargus lasgralarias* | QCAZ 11690 | MH746572 | MH746547 | Ecuador: Provincia Cotopaxi, Bosque Integral Otonga, 1950 m. |
| *Nymphargus* cf. *lasgralarias* | QCAZ 39992 | MH746573 | MH746548 | Ecuador: Provincia Carchi, Km 5 en la vía Chilma Bajo-El Placer, 2222 m. |
| *Nymphargus* cf*. lasgralarias* | QCAZ 40177 | MH746575 | MH746549 | Ecuador: Provincia Carchi, Chilma Bajo, Finca de Aníbal Pozo, 2071 m. |
| *Nymphargus* sp | KU 202796 | MH746576 | MH746550 | Ecuador: Provincia Sucumbíos, 18 km E Santa Bárbara |
| *Nymphargus* sp | KU 202801 | MH746577 | MH746551 | Ecuador: Provincia Carchi, 5 Km W La Gruel |
| *Nymphargus sucre* | MZUTI 1421 | MH746578 | MH746552 | Ecuador: Provincia Morona Santiago: Camino entre Plan de Milagro y Gualaceo, 2159 m |
| *Nymphargus sucre* | MZUTI 1422 | MH746579 | MH746553 | Ecuador: Provincia Morona Santiago: Camino entre Plan de Milagro y Gualaceo, 2159 m |
| *Nymphargus cariticommatus* | MZUTI 1417 | MH746580 | MH746554 | Ecuador: Provincia Morona Santiago: Camino entre Plan de Milagro y Gualaceo, 2159 m |
| *Nymphargus cariticommatus* | Mry 544 | MH746581 | MH746555 | Ecuador: Provincia Zamora Chinchipe: Shucos, on the old road from Loja to Zamora |
| *Nymphargus* sp | QCAZ 41572 | MH746582 | MH746556 | Ecuador: Provincia Zamora Chinchipe: río en Miazi Alto, 1282. |
| *Nymphargus* sp | QCAZ 41573 | MH746583 | MH746557 | Ecuador: Provincia Zamora Chinchipe: río en Miazi Alto, 1282. |
| *Nymphargus cochranae* | QCAZ 22196 | MH746584 | MH746558 | Ecuador: Provincia Napo: Km 13 en la vía Loreto-Coca, 1323 m. |
| *Nymphargus anomalus* | QCAZ 45702 | MH746585 | MH746559 | Ecuador: Provincia Pastaza: Río Challuwa Yaku, Reserva Comunitaria Ankaku, 1668 m |
| *Nymphargus* sp | QCAZ 41071 | MH746586 | NA | Ecuador: Provincia Napo: Volcán Sumaco, 1771 m |
| *Nymphargus* sp | QCAZ 45713 | MH746587 | NA | Ecuador: Provincia Pastaza: Río Challuwa Yaku, Reserva Comunitaria Ankaku, 1668 m |
| *Nymphargus siren* | QCAZ 30977 | MH746588 | NA | Ecuador: Provincia Napo: Río Salado |
| *Nymphargus* sp | LSB 210 | MH746589 | MH746560 | Colombia: Departamento Antioquia: Municipio de Frontino: carretera Murrí-La Blanquita |
| *Nymphargus spilotus* | JD 060 | MH746590 | MH746561 | Colombia: Departamento Caldas: Parque Nacional Selvas de Florencia, 1900-2100 m. |
